# Rectal Administration of Rifampicin and Isoniazid Suppositories: An Alternative Approach for the Treatment of Tuberculosis in a Patient with Multiple Comorbidities

**DOI:** 10.3390/life15050773

**Published:** 2025-05-12

**Authors:** Ioana Munteanu, Beatrice Burdușel, Catalin Constantin Coca, Dănuț Zisu, Constantin Gheorghevici, George Alexandru Diaconu, Diana Georgiana Stan, Nicolae Feraru, Andrei Tivda, Cristian George Popa, Florin Dumitru Mihălțan, Corina Marginean

**Affiliations:** 1 Faculty of Medicine, Titu Maiorescu University, 040441 Bucharest, Romania; ioana.munteanu2015@yahoo.ro; 2 “Marius Nasta” Institute of Pneumophtisiology, 050159 Bucharest, Romania; 3 Faculty of Medicine, Carol Davila University of Medicine and Pharmacy, 050474 Bucharest, Romania; 4 Faculty of Medicina, George Emil Palade University of Medicine and Pharmacy, Science and Technology, 540142 Târgu Mureș, Romania; corina.marginean@umf-st.ro

**Keywords:** tuberculosis, rectal suppositories, rifampicin, isoniazid, peritoneal

## Abstract

This article reports the case of a patient with a gastric neoplasm and total gastrectomy, presenting with severe digestive intolerance, who developed peritoneal and pulmonary tuberculosis. Standard treatment could not be administered. Therefore, treatment was initiated with isoniazid and rifampicin suppositories, and intravenous levofloxacin and amikacin, with significant remission of the digestive symptomatology. Although treatment with rifampicin suppositories has demonstrated efficacy in tuberculosis, it is rarely used in practice. This case highlights the importance of individualizing tuberculosis treatment and demonstrates that rectal administration of isoniazid and rifampicin suppositories, combined with intravenous levofloxacin and amikacin, was successfully used to treat tuberculosis in a patient with severe digestive intolerance, highlighting a potential alternative regimen when standard oral therapy is not feasible.

## 1. Introduction

Tuberculosis (TB) is a chronic infectious disease caused by the primary etiological agent *Mycobacterium tuberculosis*, with a predominant pulmonary tropism. The disease can manifest in various forms, ranging from latent infection to active disease, with a variable clinical presentation and the potential to develop severe complications [1]. Although mostly treatable, tuberculosis reemerged as the leading cause of death due to an infectious agent in 2023, after being surpassed by COVID-19 for three years, according to the WHO [2].

Treatment of active tuberculosis is most often carried out using standardized regimens; however, the individualization of antituberculosis therapy is justified in some cases. The most common route of drug administration is oral, including syrups for children, followed by intravenous administration [3]. Non-inferiority of rifampicin has been demonstrated with suppository administration [4], although this option is rarely used.

## 2. Case Presentation

We present the case of a 51-year-old male, a former smoker (40 pack-years, abstinent for 10 years), diagnosed with gastric neoplasm (poorly cohesive carcinoma with “signet ring” cells) one year prior to presentation to our service. The patient underwent a total gastrectomy and Roux-en-Y esophagojejunostomy and was undergoing chemotherapy. His medical history included arterial hypertension, previous myocardial infarction (severe single-vessel disease, treated with primary percutaneous coronary intervention with a stent on the left anterior descending artery), cardiorespiratory arrest due to ventricular fibrillation (resuscitated), and major right bundle branch block.

**Figure 1:**
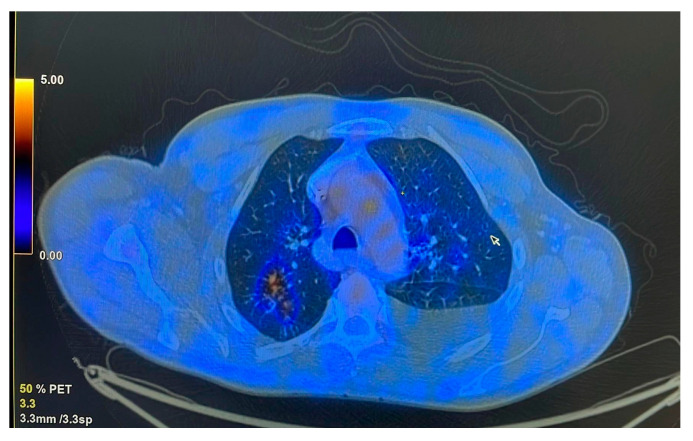
Metabolic activity in the posterior segment of the right upper lobe (RUL).

The patient was referred to the pulmonology department following combined imaging exploration (computed tomography—CT; positron emission tomography—PET) which revealed multiple pulmonary micro- and macronodular lesions with diffuse contours, located in the segments of both upper lobes and the right Fowler segment, with varying degrees of metabolic activity (Figure 1). Additionally, a flat, moderately enhancing lesion was located on the left lateral pleura, along with left pleurisy. There were also flat, metabolically active lesions at the level of the inner surface of the diaphragmatic domes and large-volume ascites.

The patient’s general condition was poor, with inappetence and a marked limitation of physical activity, with activities such as moving to the toilet being strenuous. Clinically, the patient was malnourished (BMI = 16 kg/m^2^), inappetent, and dehydrated, presenting with abdominal distension and mild diffuse tenderness on palpation. Anamnestically, the patient reported daily vomiting (fasting and postprandial) for several weeks and the absence of bowel movements for 10 days, although the patient was passing flatus.

Vital signs were within normal limits. Laboratory findings showed leukocytosis (14.91 × 10^3^/μL) with neutrophilia (79.6%), thrombocytosis (957 × 10^3^/μL), an altered ionogram with hypochloremia (77 mmol/L), hyponatremia (120 mmol/L), and elevated serum urea (97 mg/dL).

## 3. Investigations

Induced sputum and GeneXpert TB testing revealed traces of *Mycobacterium tuberculosis*, though rifampicin resistance could not be assessed. Despite difficulties related to vomiting, repeated bronchoscopy attempts were made until an adequate bronchial sample was obtained, confirming a positive GeneXpert result without evidence of drug resistance.

**Figure 2:**
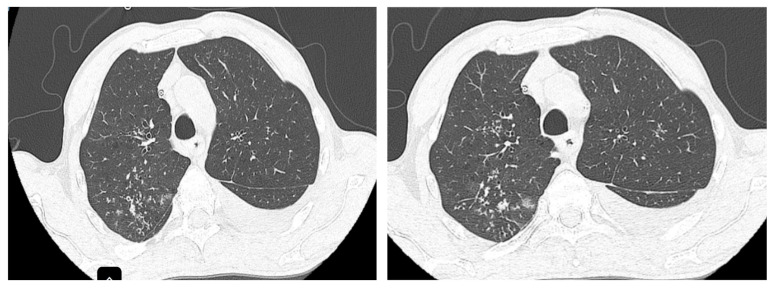
“Tree-in-bud” infiltration in the posterior segment of the right upper lobe (RUL) before treatment.

The CT scan performed at the initiation of treatment revealed micronodular “tree-in-bud” infiltration predominantly in the upper half (Figure 2), bilaterally, with left-sided pleural effusion and large-volume fluid in the abdominopelvic cavity.

Regarding the large-volume ascites and suspicion of peritoneal tuberculosis, the cytological examination revealed rare mesothelial cells and lymphocytes and very rare neutrophils, without neoplastic cellularity. Therapeutic paracentesis was performed during the first two weeks prior to starting antituberculosis treatment.

For a definitive diagnosis, invasive diagnostic investigations are necessary, with the gold standard being peritoneal biopsy. However, given the patient’s altered general condition and the positive result of the GeneXpert TB test at the pulmonary level, this procedure was not feasible, as our hospital does not have a general surgery department. Furthermore, transferring the patient to a surgery department of another hospital was not feasible in the context of a positive pulmonary GeneXpert test.

## 4. Treatment and Evolution

At the onset of therapy, the patient presented with persistent emetic episodes, with intolerance to orally administered medication. The attempt to place a nasogastric tube was complicated by esophageal mucosal laceration and self-limited hemorrhage, which required its removal. Subsequently, parenteral nutrition was initiated, and the patient maintained digestive intolerance, despite the administration of antiemetics or therapeutic combinations (metoclopramide, drotaverine, ondansetron, and octreotide).

In this case, standard therapeutic regimens for the treatment of tuberculosis, including isoniazid, rifampicin, pyrazinamide, and ethambutol, could not be administered due to the unavailability of injectable forms in our clinic and the intolerance to per os administration, i.e., an oral administration. Standard treatment protocols for tuberculosis recommend isoniazid dosages ranging from 4 to 6 mg/kg body weight and rifampicin doses between 8 and 12 mg/kg body weight [5]. Consequently, for the patient presented in this case, weighing approximately 45 kg, the indicated daily dosage corresponds to 180–270 mg of isoniazid and 360–540 mg of rifampicin. The therapeutic efficacy of antituberculosis regimens is enhanced through the utilization of fixed-dose combinations (FDCs). Within Romania, the sole FDC currently available integrates 150 mg of isoniazid with 300 mg of rifampicin [5]. This specific fixed-dose combination was selected for administration via suppositories in this case. For the formulation of the rectal suppositories, a prefabricated base composed of fatty acids and glycerol was used. After melting the base, the active pharmaceutical ingredients—isoniazid 150 mg and rifampicin 300 mg—were uniformly dispersed, and the resulting mixture was poured into molds for solidification. The treatment was supplemented by intravenous medication with levofloxacin 750 mg and amikacin 500 mg—corresponding to 10 mg/kg body weight—considering the possibility of toxicity at higher doses of amikacin and studies showing the efficacy of this dose in the treatment of tuberculosis [6].

Given that hepatotoxicity is a significant potential adverse effect of both isoniazid and rifampicin, hepatic function was monitored consistently throughout the treatment period, with results remaining within normal limits (Table 1). To mitigate the risk of isoniazid-induced peripheral neuropathy, vitamin B6 (pyridoxine) supplementation was concurrently administered. Furthermore, renal function was frequently assessed due to the potential nephrotoxicity associated with amikacin; however, no significant alterations were observed during therapy (Table 1).

Regarding parenteral nutrition, total parenteral nutrition (TPN) was administered during the initial week. However, due to its subsequent unavailability from the pharmacy, the regimen was transitioned to an intravenous administration of 500 mL of 10% glucose solution twice daily, supplemented with 500 mL of physiological saline solution per day for the following period. Oral nutritional supplementation was not tolerated by the patient prior to the initiation of antituberculosis therapy. Approximately two to three weeks after initiating the treatment, the patient gradually resumed enteral feeding, which subsequently allowed for the tapering of the intravenous glucose solution.

**Figure 3:**
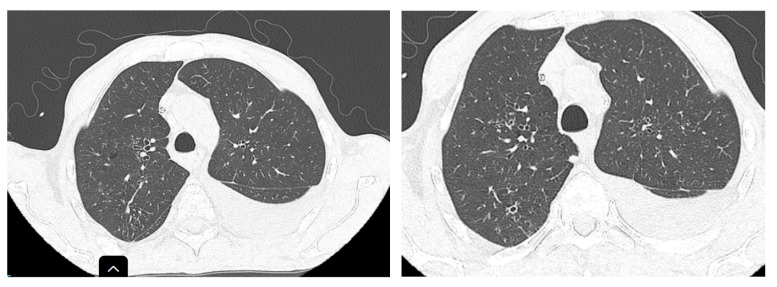
Improvement in “tree-in-bud” infiltration in the posterior segment of the right upper lobe (RUL) after 2 months of treatment.

**Figure 4:**
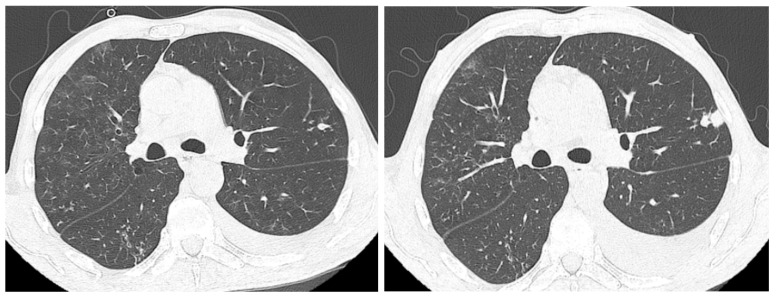
Enlargement of the pre-existing nodule and appearance of a new nodular formation at the superior lingular level.

Two months after the initiation of treatment (Figure 3), regression of interstitial densities, “tree-in-bud” micronodules, and the regression of ascites were noted. Although the lesions probably associated with tuberculosis were ameliorated, new pulmonary nodular lesions were detected, as well as an increase in a pre-existing nodule (Figure 4), most likely in the context of the underlying neoplastic pathology, whose treatment was interrupted during tuberculosis treatment.

Under the instituted treatment, the patient’s condition improved, resuming oral feeding and completing the first two months of treatment with the mentioned combination. The patient was discharged and continued at-home treatment with isoniazid and rifampicin, following the completion of the initial two-month, four-antibiotic regimen.

Although the clinical evolution regarding abdominal tuberculosis was impressive, regarding the suspicion of pulmonary tuberculosis, cultures at two months from induced sputum, on a Lowenstein–Jensen solid medium, as well as the culture from bronchial aspirate, were negative.

At the end of the 3-month hospitalization period, the patient presented a weight gain of approximately 15 kg, resolution of ascites without recurrence, significant improvement in appetite, and improvement in general condition with the resumption of physical activity, including walks and daily bicycle exercises. This favorable clinical outcome retrospectively confirmed the correctness of the therapeutic decision.

## 5. Discussions

The diagnosis of peritoneal tuberculosis can be difficult, given its nonspecific presentation and the limitations of current diagnostic methods. A high degree of suspicion, especially in patients with risk factors for tuberculosis, is crucial. For diagnosis, a combination of clinical, laboratory, imaging, and histopathological elements is often required, and sometimes, empirical treatment may be necessary in cases with high clinical suspicion but negative test results [7]. In the context of our case, a definitive diagnosis via a peritoneal biopsy was precluded by several factors. As our institution is a single-specialty pneumophthisiology clinic without surgical capabilities, the procedure could not be performed on-site. Furthermore, transfer to a surgical department at another facility was contraindicated due to the patient’s positive pulmonary GeneXpert result, posing an infectious risk, alongside concerns raised by the consulting surgeon regarding the patient’s significant perioperative risk. Nevertheless, retrospectively, we can infer that the diagnosis of peritoneal tuberculosis was likely correct, given the patient’s positive response to the instituted antituberculosis therapy, manifested by the resolution of ascites and improvement in symptoms.

The diagnosis and treatment of tuberculosis in patients with multiple comorbidities and poor nutritional status represent a challenge. In the presented case, the diagnosis was difficult to establish, given the complex clinical picture and the lack of conclusive bacteriological evidence for peritoneal tuberculosis.

The most common route of administration for antituberculosis treatment is oral. In particular situations, the administration of pharmaceutical preparations in the form of injectable solutions, infusions, or syrups may be used. The administration of syrups is especially recommended for pediatric patients under 6 years of age [3]. The option of rifampicin and/or isoniazid suppositories is rarely used but has been described. Also, considering that tuberculosis prophylaxis is carried out with isoniazid, the in vitro release of isoniazid from suppositories was studied in order to optimize the suppository dosage and form for this indication [8].

In a 2017 study on the treatment of tuberculosis, Noriyuki and Shigeatsu mentioned that they did not find significant differences between the group treated with rifampicin suppositories and the oral administration group regarding the time required for culture and smear negativity. These findings suggest that rifampicin suppositories could represent a viable therapeutic alternative for tuberculosis patients who cannot tolerate the oral administration of standard medication [4].

Given that rifampicin has low oral bioavailability and hepatotoxicity, although it is a first-line drug, mucoadhesive rectal gel was evaluated in a 2021 study on rabbits, as a tool for rifampicin administration, which significantly improved rifampicin bioavailability compared to oral suspension and solid rectal suppositories in rabbits. In addition, toxicity studies have demonstrated the ability of in situ mucoadhesive rectal gel to minimize rifampicin-induced hepatotoxicity, so studies on other routes of administration are important [9].

In our patient’s case, digestive intolerance and the impossibility of administering standardized therapy required an atypical therapeutic approach. The use of isoniazid and rifampicin suppositories, although less conventional, proved effective in this context, allowing the patient to tolerate treatment and achieve significant clinical improvement.

Although assessing serum rifampicin levels would provide valuable pharmacokinetic data regarding absorption from suppositories, the unavailability of this assay within Romania’s public healthcare system precluded such measurements. This aspect represents a significant limitation in the context of our case.

## 6. Conclusions

Although there are few reports of similar cases in the literature, there is evidence supporting the use of isoniazid and rifampicin suppositories as a viable therapeutic option in patients with digestive intolerance and contraindications for other forms of antituberculosis drug administration [4].

This case illustrates the complexity of therapeutic management in such situations and suggests that the administration of isoniazid and rifampicin suppositories can be a viable alternative when other routes of administration cannot be utilized.

Regarding peritoneal tuberculosis, its diagnosis can be challenging, given its nonspecific presentation and the limitations of current diagnostic modalities. A high degree of suspicion, particularly in patients with risk factors for tuberculosis, is crucial. For diagnosis, a combination of clinical, laboratory, imaging, and histopathological elements is often required, and sometimes, empirical treatment may be necessary in cases with high clinical suspicion but negative test results [7,10,11].

In this complex case, a treatment regimen combining the rectal administration of isoniazid and rifampicin suppositories with intravenous levofloxacin and amikacin resulted in a successful clinical outcome for tuberculosis despite severe digestive intolerance. This supports the consideration of rectally administered isoniazid and rifampicin as a viable component of therapy when standard routes are contraindicated.

## Figures and Tables

**Table 1:** Laboratory blood investigations conducted at different time points during therapy.

Blood Parameters	Initiation of Treatment	1 Month of Treatment	2 Months of Treatment
Serum creatinine RR (reference range): 0.7–1.2 mg/dL	1.29 mg/dL	0.52 mg/dL	0.554 mg/dL
Serum urea RR: 17–43 mg/dL	97.9 mg/dL	21 mg/dL	27.3 mg/dL
Alanine transaminase RR: 0–50 U/L	15.6 U/L	12.6 U/L	41.9 U/L
Aspartate transaminase RR: 0–50 U/L	16.3 U/L	19.1 U/L	27.8 U/L
Hemoglobin RR: 13.5–16.9 g/dL	13.4 g/dL	9 g/dL	11.5 g/dL
Platelet count $\mathrm{RR}:\;166–368\;\times \;10^{3}$ cells/μL	$957\;\times \;10^{3}$ cells/μL	$491\;\times \;10^{3}$ cells/μL	$413\;\times \;10^{3}$ cells/μL
Erythrocyte sedimentation rate RR: 2–20 mm/h	23 mm/h	30 mm/h	10 mm/h

## Data Availability

Data used to support the findings of this article are available from the corresponding author upon request.

## Abbreviations

The following abbreviations are used in this manuscript:

BMI	Body Mass Index
CT	Computed Tomography
FDCs	Fixed-Dose Combinations
GeneXpert TB	Refers to the Specific Diagnostic Test For Mycobacterium Tuberculosis
PET	Positron Emission Tomography
RUL	Right Upper Lobe
TB	Tuberculosis
TPN	Total Parenteral Nutrition
WHO	World Health Organization
COVID-19	Coronavirus Disease 2019
RR	Reference Range
